# Regulation of pancreatic beta-cell mass and its importance for antidiabetic therapies

**DOI:** 10.1007/s00109-026-02716-3

**Published:** 2026-09-18

**Authors:** Sigurd Lenzen

**Affiliations:** Institute of Experimental Diabetes Research, Hannover Medical School, 30625 Hannover, Germany

The proliferation rate of insulin-producing beta-cells within the pancreatic islets of Langerhans is low compared to other cell types. In rodents, it ranges from 1% to 2% per day. In humans, it is even lower, between 0.1% and 0.2% per day [for review, see 1, 2]. The rate is higher in children and adolescents than in adults, since during physical growth, beta-cell mass must expand in parallel with the increasing body mass to meet the rising insulin demand.

While the formation of new cells from progenitor cells plays a central role in expanding the beta-cell mass, particularly during the neonatal phase and early childhood [3–6], the proliferation rate is very low in adulthood [1, 2, 5, 7–11]. However, it is not zero because neoformation of beta-cells via self-duplication continues to occur [1, 2, 6, 12, 13]. Nevertheless, this low rate is sufficient to compensate for losses due to apoptotic beta-cell death, thereby maintaining a stable beta-cell population. Thus, beta-cells retain a certain degree of plasticity even in adulthood [14].

This low rate of beta-cell proliferation is not inherently problematic. The organism evidently possesses “knowledge” of a specific “set point”. This protects the individual against insulin overproduction, which can even become life-threatening due to the high toxicity of insulin. A prime example of this is persistent hyperinsulinemic hypoglycemia of infancy (PHHI), a congenital disorder in which the genetically determined regulation of the functional beta-cell mass is defective [15, 16].

Furthermore, beta-cell mass is subject to complex hormonal regulation. It is well-known that thyroid hormones reduce beta-cell mass via apoptosis, whereas glucocorticoids increase it through proliferation—a pattern observed in all mammals, including humans [17, 18]. The well-documented expansion of beta-cell mass during pregnancy also supports this view [19].

The proliferative capacity of beta-cells is particularly relevant because it can not only counteract the deterioration of the metabolic state during the pre-diabetic phase but may also support therapeutic efforts to restore metabolic control after diabetes onset. On the other hand, it is important to consistently mitigate any increased workload on the beta-cells such as caused by obesity or autoimmune attack since it always carries the risk of deteriorating beta-cell function and reducing beta-cell mass.

Results from animal studies indicate that the propensity for compensatory beta-cell proliferation increases when beta-cell mass has declined due to disease [20]. As therapeutic options become increasingly diverse, this issue will gain significance in the future. In particular, the question arises as to whether pharmacotherapeutic interventions can contribute to restoring reduced beta-cell mass beyond endogenous, genetically driven proliferation. After all, both T1DM and T2DM are associated with a progressive beta-cell loss over time [21–24]. In this context, determining the optimal timing for such therapeutic interventions is of crucial importance.

## Significance of the decline in beta-cell mass with respect to the development of type 2 diabetes (T2DM) and the potential to restore beta-cell mass

In many adults, pancreatic beta-cell mass increases by up to 50% between the third and fifth decades of life, coinciding with the development of obesity during this period [8, 25]. This expansion of beta-cell mass is driven by increasing insulin resistance in association with a Western diet and lifestyle and a concomitant rise in insulin production [25]. Only a minority—no more than 20% of individuals—are able to maintain this elevated beta-cell mass and the resulting adequate insulin supply to the body into old age, despite obesity [26]. The vast majority fails to do so. Thus following this phase of life, a gradual decline in beta-cell mass leads to insulin deficiency and, consequently, the development of T2DM [25].

For this reason, hormones capable of contributing to an increase in beta-cell mass have moved to the forefront of scientific interest. In this context, the gut hormone glucagon-like peptide-1 (GLP-1) is particularly noteworthy. A study by Erbasan et al. [27], recently published in this journal, has provided further compelling evidence in this regard. Gene therapy in neonatal and adult streptozotocin-diabetic rats involving GLP-1 release after lentivirally mediated overexpression of this incretin hormone in the hepatocytes of these animals [27] supports pancreatic beta-cell regeneration. Thus the study demonstrates that plasticity—and the associated potential for beta-cell neogenesis from progenitor and duct-derived cells—is high in the neonatal pancreas. In contrast, generation of new beta-cells in the adult pancreas occurs mainly through self-duplication of existing beta-cells [27] confirming earlier observations [12], with limited potential for neogenesis and, consequently, for recovery of beta-cell mass. Therapy with GLP-1 therefore represents a future option to counteract the insulin deficiency that develops gradually during the course of T2DM due to progressive beta-cell failure. Ideally, this intervention should occur before metabolic deterioration. Treatment with a GLP-1 analogue would not only support the preservation of beta-cell mass but also promote weight loss [28].

## Reduction of beta-cell mass during the development of autoimmune type 1 diabetes (T1DM) and possibilities for a sustained increase within the scope of future therapeutic options with curative potential

This presents a significant challenge. Since over 90% of patients who develop T1DM resulting from autoimmune-mediated beta-cell destruction [29, 30] have no family history of this disease, almost all individuals are diagnosed only when they manifest overt T1DM at stage 3 of the disease [31]. Interestingly, however, in the weeks following disease onset, most of these patients enter a remission phase while receiving insulin replacement therapy during which the remaining beta-cells recover [31]. It is during this phase—ideally following beta-cell recovery but prior to the onset of renewed metabolic deterioration when the recovery wanes [31] —that the chances of achieving a significant increase in beta-cell mass through self-duplication of existing beta-cells are optimal.

However, this requires the availability of antidiabetic therapies with curative potential. As numerous monotherapies have proven ineffective in this regard, only combination therapies are promising candidates [30, 32]. These should be based on a profound understanding of the underlying pathomechanisms [30] and on reliable preclinical animal studies to facilitate successful translation to patients [32]. However, successful T1DM combination therapies require the presence of at least 30% of normal beta-cell mass at the start of treatment [30].

Based on generally accepted beta-cell proliferation rates—1% per day in rats and 0.1% per day in humans [1, 2]—it can be predicted that, following a cessation of autoimmune-mediated beta-cell destruction, a period of approximately two months is required in rats to increase beta-cell mass from one-third to two-thirds of the original mass found in a healthy state. This aligns with experimental results from animal models [30]. By analogy, the time required for this increase in beta-cell mass in humans would be one to two years. A recent medical case study found that a combination of the two therapeutic antibodies teplizumab and adalimumab has proven effective in patients, resulting in sustained metabolic health one year after the start of therapy—with no symptoms of diabetes and no need for insulin replacement [33].

Only after successfully suppressing the autoimmune-mediated destruction of beta-cells—driven by proinflammatory cytokines (specifically TNFα and IL-1β)—and eliminating the immune-cell infiltration within the islets following such combination therapy for T1DM [30, 32, 33], does the administration of an agent such as an GLP-1 analogue become a viable option to enhance spontaneous beta-cell regeneration. This approach is particularly suitable when beta-cell mass is already significantly reduced (< 50%), as it provides supplementary support for beta-cell proliferation. Administering the agent earlier, while the autoimmune attack is still ongoing, would merely result in the immediate destruction of the beta-cells newly formed through proliferation. Studies in animal models have shown that, during an ongoing autoimmune attack, proliferation does attempt to counteract beta-cell loss, but unsuccessfully [30].

## Conclusion

Despite the different underlying pathomechanisms responsible for beta-cell loss—and the resulting insulin deficiency that leads to the development of T1DM and T2DM—distinct “windows of opportunity” exist for therapeutic interventions at appropriate stages during the course of both diseases. This includes the possibility of supporting endogenous mechanisms for increasing beta-cell mass through beta-cytotrophic molecules.

## Data Availability

Sources of all data analyzed and discussed in this article are cited therein as references.

## Abbreviations

T1DM: type 1 diabetes mellitus
T2DM: type 2 diabetes mellitus
GLP-1: glucagon like peptide-1
